# Psychosocial and career outcomes of peer mentorship in medical resident education: a systematic review protocol

**DOI:** 10.1186/s13643-017-0571-y

**Published:** 2017-08-31

**Authors:** Helen Pethrick, Lorelli Nowell, Elizabeth Oddone Paolucci, Liza Lorenzetti, Michele Jacobsen, Tracey Clancy, Diane L. Lorenzetti

**Affiliations:** 1 Department of Community Health Sciences, Cumming School of Medicine, University of Calgary, 3280 Hospital Drive NW, Calgary, AB T2N 4Z6 Canada; 2 Faculty of Nursing, University of Calgary, 2500 University Drive NW, Calgary, AB T2N 1N4 Canada; 3 Faculty of Social Work, University of Calgary, 2500 University Drive NW, Calgary, AB T2N 1N4 Canada; 4 Werklund School of Education, 2500 University Drive NW, Calgary, AB T2N 1N4 Canada

**Keywords:** Burnout, Medical education, Medical residents, Mental wellbeing, Mentorship, Peer mentors, Systematic review

## Abstract

**Background:**

Many medical residents lack ready access to social and emotional supports that enable them to successfully cope with the challenges associated with medical residency. This absence of support has been shown to lead to high levels of burnout, decreased mental wellbeing, and difficulty mastering professional competencies in this population. While there is emerging evidence that peer mentoring can be an important source of psychosocial and career-related support for many individuals, the extent of the evidence regarding the benefits of peer mentorship in medical residency education has not yet been established. We describe a protocol for a systematic review to assess the effects of peer mentoring on medical residents’ mental wellbeing, social connectedness, and professional competencies.

**Methods:**

Studies included in this review will be those that report on peer-mentoring relationships among medical residents. Quantitative, qualitative, and mixed-methods studies will be eligible for inclusion. No date or language limits will be applied. We will search EMBASE, MEDLINE, PsychINFO, Web of Science, Scopus, ERIC, Education Research Complete, and Academic Research Complete databases to identify relevant studies. Two authors will independently assess all abstracts and full-text studies for inclusion and study quality and extract study data in duplicate.

**Discussion:**

This is the first systematic review to explicitly explore the role of peer mentoring in the context of medical residency education. We anticipate that the findings from this review will raise awareness of the benefits and challenges associated with peer-mentoring relationships, further the development and implementation of formal peer-mentoring programs for medical residents, and, through identifying gaps in the existing literature, inform future research efforts.

**Systematic review registration:**

This protocol has not been registered in PROSPERO or any other publicly accessible registry.

**Electronic supplementary material:**

The online version of this article (10.1186/s13643-017-0571-y) contains supplementary material, which is available to authorized users.

## Background

Medical residents, in Canada and internationally, suffer from higher levels of burnout, stress, and depression compared to the general population [1–5]. While terminology varies globally, for the purposes of this review, medical residents are defined as medical doctors currently involved in post-graduate training under the supervision of attending or senior physicians. Residents’ mental wellbeing can directly impact patient care, and residents who have experienced symptoms of burnout also report lower quality patient care, including increases in medical errors [6]. Burnout in medical residents can arise from stressful work conditions, such as high workload, low autonomy in the workplace, limited financial remuneration, and a lack of sense of community among colleagues [4]. Medical professionals and regulatory bodies have proposed placing limits on resident work hours as a means of improving patient care and addressing mental wellbeing concerns among medical residents [4, 7, 8]. However, researchers observed no demonstrated improvements in resident wellness after resident work hour restrictions were mandated by the Accreditation Council for Graduate Medical Education in the United States; further, both patient outcomes and residents’ exam performance worsened [8]. If current strategies are ineffective in improving residents’ job performance and wellbeing, then it is critical to consider other factors and approaches [9].

### Medical residents’ mental wellbeing

Many factors can contribute to high levels of burnout among medical residents. The transition from undergraduate medical education to first-year medical residency can be a challenging period of adjustment for many residents. First-year medical residents are expected to assume new and greater responsibilities and workloads as compared with their undergraduate counterparts. They may have limited opportunities to interact with and obtain support from their peers and are typically assigned to work with diverse teams of nurses, senior residents, social workers, supervisors, and other medical personnel. Research suggests that many medical residents believe that they are perceived by other team members as lacking in experience and knowledge, and are thus accorded little support or respect [10–12]. Indeed, many report that supervisors, in particular, do not provide adequate levels of emotional support [11, 12]. Studies examining the relationship between social or emotional support and burnout in comparable populations (college students, social workers, and counselors) have found that psychosocial support and burnout are inversely related [13–15]. In 2002, a randomized controlled trial evaluated the effects of peer-group participation on healthcare workers’ self-reported health outcomes, levels of burnout, and “perceived changes in work conditions” [16]. This study found that participation in peer-support groups significantly increased perceptions of overall health (*p* = 0.01) and reduced perceptions of increased work demands (*p* = 0.014) [16]. Qualitative findings further revealed that participants with access to peer support experienced greater levels of work-based support, decreased stress and anxiety, and increased self-confidence and sense of belonging [16].

### Core competencies in medical resident education

In recent years, there has been a trend towards incorporating competency-based frameworks into medical residency education. This represents a shift away from educational frameworks that prioritize time-dependent curriculum and minimal disruption to hospital systems over developing residents’ competence in essential skills [17–19]. Competency-based medical education focuses on furthering the development of core competencies that physicians, as medical experts, should exemplify in practice: communication, professionalism, scholarship, health advocacy, collaboration, leadership, and medical expertise [19, 20]. Curriculums that focus on core competencies can enable the development of well-rounded, capable medical professionals who are sufficiently equipped to deal with increasingly complex medical systems and diverse patient needs [17, 20].

Research on mentorship suggests that peer mentors can further the development of many core medical competencies essential to medical residents’ education including professionalism, communication, and collaboration [20–24]. Professionalism encompasses a commitment to patients, society, and the medical profession [19]. It focuses on the formation of professional identity, collegiality, supportive attitudes towards colleagues (especially those in need), and the development of a sense of responsibility to colleagues in the form of peer assessment and mentorship [19]. Communication and collaboration are competencies closely related to professionalism, emphasizing the ability of physicians to work effectively and supportively with other members of health care teams, show respect towards colleagues, and share knowledge within the medical profession [19]. These specific core competencies are largely formed by ongoing practice in the medical profession, rather than in-class instruction [20, 25]. Mentoring, with its emphasis on the provision of psychosocial and career-related supports, has the potential to reduce resident burnout, increase socialization, and further the development of core professional competencies [13–15, 20–22].

### Peer mentorship: a contributor to medical resident education

Mentoring, in a professional context, is characterized as an ongoing supportive developmental relationship between a more experienced professional and a less experienced newcomer [26]. Mentors can provide a variety of psychosocial (friendship, role modeling, and identity development) and career-related (organizational socialization and professional advice) supports [21]. Through the transmission of tacit organizational knowledge and explicit practical advice, mentoring relationships enable newcomers to develop essential career competencies and socialize into their professional environments [22]*.*

Early developmental theories conceptualize mentoring as a hierarchical and unidirectional mentor-mentee relationship [22]*.* However, an alternative theoretical understanding of mentoring, supported by social and learning theories, emphasizes the reciprocity and co-learning that can characterize mentoring experiences [22]. In peer-mentoring relationships, mentor-mentee pairs are at similar stages in their careers or education. As such, peer mentors may be able to provide greater degrees of emotional and practical support than more senior mentors [24]. Peer-mentoring relationships deemphasize hierarchical power differentials that can exist between mentors and mentees, and emphasize the mutual benefit that participants can derive from these relationships [22, 24]*.*

In studies involving comparable populations in educational settings, peer mentoring has been shown to provide participants with a variety of developmental and psychosocial benefits, enabling them to better adapt to their learning environments [27–30]. Authors of a systematic review of mentoring in academic nursing found that, in general, peer-mentoring programs enabled nurses to develop collaborative peer relationships that reduced self-perceived stress [30]. Among healthcare professionals, and medical residents in particular, peer-mentoring relationships can increase social support and reduce symptoms of burnout [12, 16, 31–34]*.* In 2007, the authors of a study of peer relationships among medical residents in the Netherlands reported that peer support was positively associated (*r* = 0.18, *p* < 0.05) with reductions in self-reported burnout [12].

Peer mentorship can also further the development of medical residents’ work-based professionalism, collaboration, and communication competencies [22, 25, 32]*.* Studies involving medical residents have reported that peer mentors enable the development of key professional competencies such as communication, listening, and research skills [32]. These findings are further supported by research conducted with graduate students in academic settings. An evaluation of a peer-mentoring program for graduate counseling students demonstrated that peer mentoring enhanced professional development and professional identity formation for both mentors and mentees; mentees benefited from their mentors’ wealth of experience; and mentors practiced developing professional boundaries within collegial relationships [23]. In another study, graduate students in a peer-mentoring program also reported that they were able to better navigate the organizational norms and politics of their learning environments, and found more success in developing positive professional relationships [28].

While prior reviews have addressed mentoring among physicians, junior doctors, and medical students, there has, to date, been no explicit systematic review of peer mentoring among medical residents [35–38]. Our review will address an important gap in the literature by comprehensively assessing the impact that peer mentors can have on reducing burnout, enhancing overall emotional wellbeing, and furthering the development of professional competencies in medical residency education.

### Aim

The objective of this systematic review is to explore how peer mentorship relates to psychosocial and learning outcomes among medical residents. The systematic review question is: how does peer mentorship affect medical residents’ mental wellbeing, social connectedness, and professional competencies?

## Methods/design

The development of this systematic review protocol has been informed by systematic review guidelines published by York University’s CRD (Centre for Reviews and Dissemination) [39]. This protocol does not focus on health conditions and health-related outcomes; it therefore is not eligible for and thus has not been registered with PROSPERO.

### Eligibility criteria

**Fig. 1:**
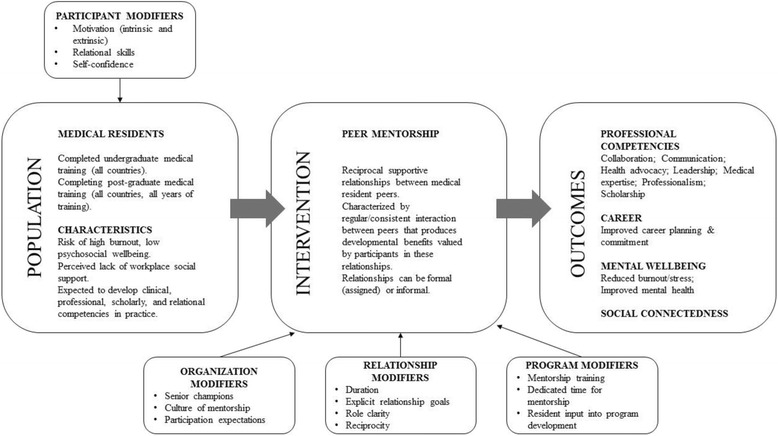
Logic model demonstrating the assumed relationship between peer mentorship and relevant career and psychosocial outcomes among medical residents

The question of relevance is: how does peer mentorship impact medical residents’ mental wellbeing, social connectedness, and medical professional competencies? We developed an a priori logic model as a visual representation of our assumptions regarding the nature, progress, modifiers, and anticipated outcomes of these relationships in the context of medical residency education (Fig. 1). This model will inform the progress of this review.

#### Participants

Studies will be included if they involve medical residents. Medical residents are medical doctors currently involved in post-graduate training under the supervision of attending or senior physicians. We will exclude studies where the study population is non-medical residents (undergraduate medical students, nursing residents, clinicians, post-doctoral fellows, or faculty). We will only include those studies where medical resident data are reported separately from that of other participants. We will contact corresponding authors for verification in cases where it is unclear if study participants meet our inclusion criteria.

#### Interventions

Studies that report on formal peer-mentoring programs or peer-support relationships among medical residents will be included. Interventions that identify mentors as clinicians, supervisors, community mentors, or peers who are not medical residents will be excluded.

#### Outcomes

Prior literature on medical resident education, and peer mentoring in the contexts of graduate medical, and nursing education informed the identification of outcomes relevant to this review [2, 17, 19, 27, 28, 30]. Studies will be included if they report on one or more of four categories of outcomes: (1) professional competencies (clinical skills, collaboration, communication, health advocacy, leadership, professionalism, scholarship); (2) career (career planning or commitment); (3) mental wellbeing (burnout, stress, overall mental health); or (4) social connectedness (shared purpose, sense of community, emotional support, friendship). We will also extract data on any other outcomes of medical residents’ peer-mentoring or peer-support relationships reported in studies identified in this review.

#### Study type

Any study design or approach (e.g., qualitative, quantitative, or mixed methods) will be included to encompass the widest range of studies possible. We will not exclude any studies based on language, publication date, or geographic location.

### Information sources and search strategy

We will search databases that index journals related to medical research or education research including MEDLINE, EMBASE, Scopus, Web of Science, PsychINFO, ERIC, Education Research Complete, and Academic Research Complete from their inception to present. The search will include four main concepts: (1) peers; (2) mentorship; (3) medicine; and (4) residents. Keywords associated with these concepts will be searched as database-specific subject headings (where applicable) and title/abstract words (Table 1). This search strategy has been developed in consultation with a research librarian. We will scan the reference lists of all eligible studies to identify additional studies of relevance to this review. We will compile all of the search results in Endnote™ v8 and remove any duplicates prior to study selection. The search for relevant literature will be updated within 90 days of publication of this review.

**Table 1:** Provisional search strategy for Ovid MEDLINE

Search strategy
1. Mentors
2. (mentorship or mentor or mentors or mentoring or mentee* or protege*).tw,kw
3. 1 or 2
4. exp “Internship and Residency”/
5. Education, Medical, Graduate/
6. (fellow* or junior doctor* or house staff or housestaff or house officer* or registrar*).tw,kw
7. ((anaesthesiolog* or anesthesiolog* or emergency medic* or family med* or general med* or geriatric* or gynaec* or gynec* or internal med*or neurolog* or obstetric* or paediatric* or pediatric* or psychiatr* or radiolog* or special* or surg*) adj10 (trainee* or training or resident* or residents)).tw,kw.
8. ((intern* or resident* or residenc*) adj10 (medical or medicine)).tw,kw
9. (((graduate or postgraduate or post-graduate) adj10 (doctor* or medical or medicine)) and (educat* or train*)).tw,kw.
10. 4 or 5 or 6 or 7 or 8 or 9
11. Peer group/
12. (peer* or buddy or buddies).tw,kw.
13. 11 or 12
14. 3 and 10 and 13

### Study selection

Studies identified through searches will be assessed for inclusion or exclusion in two phases. In the first phase, two authors (HP, DLL) will independently screen the study abstracts in duplicate. We will calculate a Kappa statistic to assess the reliability of inter-rater agreement for the abstract screening process [40]. Discrepancies will be resolved through consensus or full-text review. In the second phase, we will retrieve the full-text articles of all the included abstracts. Two authors (HP, DLL) will independently screen the full-text articles for eligibility, discarding any studies that meet any of the exclusion criteria or do not meet the inclusion criteria outlined above. We will resolve any discrepancies through consensus or consultation with a third author (LN).

### Assessment of methodological quality in individual studies

Two authors (HP and DLL) will assess the quality of the eligible studies using a variety of quality assessment tools. We will use the CASP (Critical Appraisal Skills Programme) qualitative study checklist to assess qualitative studies, the JBI (Joanna Briggs Institute) cross-sectional checklist for cross-sectional studies, the Downs and Black checklist for non-randomized quantitative studies, and the Cochrane’s Risk of Bias tool for randomized control trials [41–44]. While we will report on the results of this quality assessment, studies will not be excluded on the basis of quality alone.

### Data extraction

We will follow the University of York CRD guidelines for data extraction [39]. Data from quantitative and qualitative studies will be extracted separately. We will use spreadsheets to extract data from each of the eligible studies (Table 2). Two authors will pilot test the data extraction spreadsheets on a sample of five included studies to ensure consistency in interpretation and application. The data to be extracted will include basic study information (authors, publication date, and country of origin); study design, study objectives, and participant characteristics (program year, gender, and sample size); peer mentor program descriptions (setting, type of relationship, program length, design, and implementation); outcome measures; and study findings. Findings will include reported effects of peer mentoring (and peer-mentoring programs) on professional competencies (communication, professionalism, scholarship, health advocacy, collaboration, leadership, and medical expertise); career (career planning and commitment); mental wellbeing (including burnout); and social connectedness. One author (HP) will complete the data extraction forms for all the eligible studies, and another author (DLL) will review all data for consistency and accuracy. Studies that have been published in duplicate will be retained and assessed in full text; the most comprehensive study will be included. Any disagreements will be resolved through discussion and consensus or consultation with a third author (LN).

**Table 2:** Data extraction categories for eligible studies

Category	Data extracted
Basic information	Study authors, publication date, and country of origin
Study characteristics	Aim and objectives of study, study design, recruitment strategy, and inclusion/exclusion criteria (if relevant)
Participant characteristics	Number of participants, age ranges, gender, residency year, program and location, sample size, and any other relevant characteristics
Description of peer-mentoring program or peer support	Description of peer-mentoring program initiation, implementation, and evaluation; description of peer-support relationships; length of program; and any other individuals or groups involved
Outcome measures	Measures or tools used to gather outcomes data (chart reviews, resident evaluations, interviews, focus groups, surveys)
Reported outcomes	Professional competencies (clinical skills, collaboration, communication, health advocacy, leadership, professionalism, scholarship)
	Social connectedness (shared purpose, sense of community, emotional support, friendship)
	Psychosocial wellbeing (burnout, stress, overall mental health, self-esteem, self-efficacy)
	Mentoring relationship satisfaction (quality, retention)
	Career (perspectives, prospects, commitment)

### Synthesis of included studies

If sufficient homogenous quantitative data is present in the eligible studies, we will pool quantitative data for meta-analysis and qualitative data for narrative synthesis. However, previous reviews have found the mentoring literature to be highly heterogeneous in nature, which typically precludes meaningful meta-analysis of quantitative data [29, 30, 38]. Rather, we will apply thematic analysis and synthesis techniques to transform and integrate findings from qualitative, quantitative, and mixed-methods studies into a synthesis of convergent themes [45]. In thematic analysis, researchers use open and analytical coding techniques to analyze findings from individual studies, and gradually transform codes into broad, high-level concepts or themes [46, 47]. Convergent syntheses are those in which findings from quantitative and mixed-methods studies are transformed into qualitative themes, and integrated with findings from qualitative studies into common themes [45]. Themes in this review will be presented in a convergent narrative synthesis and, visually, in a series of tables and figures.

The reporting of this review will be guided by the PRISMA (Preferred Reporting Items for Systematic Reviews and Meta-Analyses) statement [48]. A completed PRISMA-P (Preferred Reporting Items for Systematic Review and Meta-Analysis Protocols) checklist has been submitted as an additional file to this protocol (Additional file 1). The selection of studies (identification, screening, eligibility, and inclusion) for the review will be reported using the PRISMA flowchart [48]. The flowchart will report the total number of studies initially identified in database and other searches, the number of studies remaining after duplicates were removed, and the number of abstracts and full-text studies screened and retained. Study data will be reported in a table [39, 48]. The table will include for each study (1) a citation, (2) the study design, (3) the population of the study and location or setting, (4) a description of the peer-mentoring program or mentoring relationships, (5) peer-mentoring program or relationship outcomes, and (6) any methodological limitations identified through quality assessment. The narrative section will report the number and characteristics of studies eligible for the review, highlight important findings from included studies, and describe how the studies address outcomes of interest (residents’ academic/career skills, mental wellbeing, and social connectedness). Provided that sufficient data exists, we will consider conducting subgroup analyses by study design, country, elements of program design, participant specialization, and/or year of training. In our “Discussion” section, we will briefly describe the main findings, strengths, and limitations of this review and contextualize our findings within the broader literature on peer mentorship and medical resident education [49]. We will outline the implications of our review for practice in medical resident education, and identify areas for future research, whether elaborating on current findings or addressing identified gaps in the literature.

### Validity and reliability

The systematic review team will ensure that the review process is rigorous and that the findings presented accurately reflect the relevant literature. The team includes systematic review methodologists, a research librarian, and three knowledge experts with a research focus on mentorship. All selection, appraisal, and extraction forms will be pilot tested by the reviewers to ensure consistency and reliability in their application.

## Discussion

To our knowledge, no systematic reviews of peer mentorship among medical residents currently exist in the published literature. Peer mentorship has been shown to positively contribute towards comparable populations’ learning by furthering professional identity development, providing psychosocial support, and contributing guidance on organizational norms [23, 27, 28]. However, the absence of a systematic review appraising and synthesizing the state of the evidence on peer mentoring among medical residents restricts the ability of the medical education community to consider the value of peer mentorship and assess approaches to formally integrate peer mentorship into medical residency education.

This review will further research on peer mentorship in medical resident education. By identifying gaps in the existing literature, and reporting on the impact of peer mentorship on key psychosocial and educational outcomes of relevance to residency education, this review will further the development of, and ongoing improvements to, peer-mentoring programs in the context of medical residency education.

### Limitations

Similar reviews, focused on mentoring programs in undergraduate medical education and academic medicine, have found a small number of eligible studies of varying study designs, study quality, and reported outcomes [29, 30, 39]. The expected heterogeneity of the literature on peer mentorship in medical residency education, and a potentially limited availability of empirical studies, may constrain our ability to draw clear and consistent conclusions from the literature. Regardless of study heterogeneity, this systematic review will, at a minimum, provide clarity regarding the existing evidence on peer mentorship for medical residents and identify areas for future research.

## Additional file

Additional file 1: PRISMA-P 2015 Checklist. (DOCX 75 kb)

## Abbreviations

CASP: Critical Appraisal Skills Programme Oxford UK
CRD: Centre for Reviews and Dissemination, University of York
JBI: Joanna Briggs Institute, University of Adelaide
PRISMA: Preferred Reporting Items for Systematic Reviews and Meta-Analyses
PRISMA-P: Preferred Reporting Items for Systematic Review and Meta-Analysis Protocols
